# Editorial: Metabolomics as a tool in ethnobotany-driven drug discoveries

**DOI:** 10.3389/fphar.2022.1066875

**Published:** 2022-10-28

**Authors:** Nokwanda Makunga, Guoyin Kai, Elwira Sieniawska

**Affiliations:** ^1^ Department of Botany and Zoology, Stellenbosch University, Stellenbosch, South Africa; ^2^ Laboratory of Medicinal Plant Biotechnology, College of Pharmaceutical Sciences Zhejiang Chinese Medical University, Hangzhou, China; ^3^ Department of Natural Products Chemistry, Medical University of Lublin, Lublin, Poland

**Keywords:** bioactivity, natural products, bioinformatics, drug leads, pharmacology

Ethnobotany-driven drug discoveries is an ongoing challenge aiming to prove well known traditional applications of medicinal plants. The classical activity screening is nowadays more frequently replaced by efficient and conclusive metabolomics-bioinformatics based approach. Metabolomics is a dynamic and rapidly evolving field that provides insights into primary and secondary metabolites present in any living organism. It creates unique chemical fingerprints of the cell metabolism and sheds light about pathological disease conditions, progress of treatment options, establishment of new therapies or validation of traditional treatments. Typical hyphenated separation and detection techniques applied in metabolomic studies are GC-MS, LC-MS, LC-NMR, or GC-NMR, which are often followed by computational analysis and data processing using different statistical tools. The modern research platforms that are now available to researchers enable a fast and reliable determination of mechanisms of action behind plant extracts, the monitoring of diseases biomarkers and last but not least the selection of active principles in crude plant extracts or herbal mixtures. With a growing number of studies providing evidence on the activity of natural remedies from ethnobotanical resources, the field ethnopharmacology is becoming an important and decisive area of interest. Experimental assessments of medicines used in traditional knowledge need to be coupled with the use of modern chromatographic and spectroscopic techniques that are supported with bioinformatic software. Such an integrated approach is an important step when aiming to bridge the gap between tradition and science.

The studies collected within Research Topic explored disease profiles in patients after the treatment with traditional herbal composition (Ma et al.), mechanism of action of traditional herbal composition combined with classical treatment (Li et al.), metabolomic profiles of herbal extracts in relation to their protective action in human disease (Liu et al.; Salem et al.), and active biomarkers in herbal extracts (Emamzadeh et al.) Plant Chinese traditional medicines were a subject of two studies, which focused on the determination of metabolites affected by the treatment. Ma et al. evaluated the effects of Zuojin pil, a well-known formula used from 15th century in digestive system diseases. The metabolomic analysis revealed significantly changed levels of dozen of metabolites as well as inflammatory factors (COX-2, IL-4, and IL-17) suggesting that Zuojin pil acts as an inflammatory suppressor to regulate comprehensive metabolism disorders. Li et al. discussed the anti-platelet molecular mechanism of action of several Blood-activating Chinese botanical drugs. Besides proteomics and transcriptomics, metabolomics provided methodology to describe platelets as biomarkers and targets for diagnosis and treatment of cardiovascular diseases.

With the application of metabolomics Salem et al. explained the better antihypertensive effect of *Hibiscus sabdariffa* L. calyces extract obtained with hot water over the cold extraction. UPLC–MS/MS analysis traced high quantities of N-feruloyltyramine, caffeoylshikimic acid, dicaffeoylquinic acid, delphinidin-3,6″-p-coumarylglucoside, kaempferol-7,6″-p-coumarylglucoside, and myricetin which presence contributed to high bioactivity observed in animals. Similarly, Liu et al. investigated the effectiveness of Lacquer oil from the drupes mesocarp of *Toxicodendron vernicifluum* in inflammation and postpartum depression. Using metabolomics, they found 57 chemical markers discriminating black lacquer oil and white lacquer oil, of which 17 potential biomarkers have been declared to possess anti-inflammatory and/or antidepressant activities determined *in vitro* and *in vivo*. Also anti-HIV biomarkers were described in NMR-based metabolomic investigation. Emamzadeh et al. screened 57 *Helichrysum* species and performed OPLS-DA and hierarchical cluster analyses to correlate phytochemical composition and biological activity of the samples. The chlorogenic acids, compounds with cinnamoyl functional groups, and quinic acid were the most prominent compounds in the *Helichrysum* species with anti-HIV activity.

The presented research, not only confirmed the usefulness of metabolomics in determination of the mechanism of action of traditionally used formulations, but also identified the active principles responsible for the activity. Moreover, metabolomics was shown as complementary to other “omics”, underlining the power of comprehensive, systematic approach, which is currently realized within systems biology.

